# Biofilm development of *Candida boidinii* and the effect of tyrosol on biofilm formation

**DOI:** 10.1007/s10529-023-03432-5

**Published:** 2023-10-13

**Authors:** Rita Márton, Blanka Nagy, Mónika Molnár

**Affiliations:** Budapest University of Technology and Economics Department of Applied Biotechnology and Food Science, Műegyetem rkp. 3, 1111 Budapest, Hungary

**Keywords:** Biofilm formation, *Candida boidinii*, Quorum sensing, Tyrosol

## Abstract

**Objectives:**

The applicability of a simple and high-throughput method for quantitative characterization of biofilm formation by *Candida boidinii* was tested in order to evaluate the effects of exogenous tyrosol on yeast growth and biofilm formation capacity.

**Results:**

Significant concentration-, temperature and time-dependent effect of tyrosol (2-(4-hydroxyphenyl)ethanol) was demonstrated, but it differentially affected the growth and biofilm formation (characterized by crystal violet staining and XTT-reduction assay) of *Candida boidinii*. Testing biofilm based on metabolic activity displayed sensitively the differences in the intensity of biofilm in terms of temperature, tyrosol concentration, and exposure time. At 22 °C after 24 h none of the tyrosol concentrations had significant effect, while at 30 °C tyrosol-mediated inhibition was observed at 50 mM and 100 mM concentration. After 48 h and 72 h at 22 °C, biofilm formation was stimulated at 6.25–25 mM concentrations, meanwhile at 30 °C tyrosol decreased the biofilm metabolic activity proportionally with the concentration.

**Conclusions:**

The research concludes that exogenous tyrosol exerts unusual effects on *Candida boidinii* growth and biofilm formation ability and predicts its potential application as a regulating factor of various fermentations by *Candida boidinii.*

**Supplementary Information:**

The online version contains supplementary material available at 10.1007/s10529-023-03432-5.

## Introduction

Yeasts play an enormous role in our life, being responsible for principal ecosystem services through organic decomposition and fermentation. These microorganisms are important for the food industry including winemaking (Gonzalez and Morales 2022), ethanol and EPS production (Kieliszek et al. 2017) and other alcoholic beverage fermentation (Cousin et al. 2017), baking industry (Struyf et al. 2017) and several food manufacturing processes including coffee, chocolate, or table olives production (Maicas 2020). On the other hand, several pathogens are encountered among yeasts that can cause many diseases, from mild discomfort to serious illnesses. Yeast infections are caused most often by several species of *Candida* and some other pathogens like *Cryptococcus, Trichosporon* and *Rhodotorula (*Astvad et al. 2018*).* Due to the broad taxonomic boundaries of the genus *Candida*, the biotechnological application of these yeasts is also growing. Metabolically engineered *Candida famata* is capable of riboflavin (vitamin B_2_) oversynthesis and secretion into the culture medium. Even though this method needs improvement, it has great potential for industrial application (Fedorovych et al. 2021). Citric acid, mainly used in the pharmaceutical, cosmetic, and food sectors, can be synthesized by some *Candida* spp. like *C. oleophila* or *C. zeylanoides* (Anastassiadis et al. 2002; Sayın Börekçi et al. 2022). *Candida* species like *C. glycerinogene* can also play a role in ethanol fermentation from lignocellulose (Zhao et al. 2019), and this multistress tolerant species has been successfully used also for glycerol production (Zhuge et al. 2001).

Among *Candida* species, *Candida boidinii* is a less known and researched yeast, though it has great biotechnological importance. *C. boidinii* is also widespread and it has been isolated from various products of food processes such as wine fermentations, olive manufacturing, and natural environments (soil, seawater, sugar-rich tree species, etc.). *C. boidinii* has also been reported as one of the most suitable yeasts for xylitol production (Vongsuvanlert and Tani 1989; Fehér et al. 2015; Romero-García et al. 2022).

Additionally, *Candida* species are currently the subject of research from several other perspectives. Among these, studying quorum sensing -mainly with *Candida albicans-* is of particular importance. Quorum sensing (QS) is a cell-to-cell communication that makes possible for microorganisms to modify their behaviour in response to changes in population density. Quorum sensing enables the microbial population to behave as a multi-cellular organism and it can be important during host colonization, adaptation to changing environment through biofilm formation. Bacterial quorum sensing has been widely studied for a long time, but the fungal intercellular communication has only been described in recent years (Albuquerque and Casadevall 2012). However, according to several studies, QS is also correlated to biofilm formation in fungi similar to prokaryotes. Biofilm formation is one of the most important virulence factors of the opportunistic pathogen bacterial and fungal species. Biofilms are multicellular communities of microorganisms that adhere to a wide variety of surfaces to provide adequate living space for the cells and protect them from harmful factors.

The microbial communication is based on the exchange and sensing of low-molecular-weight signal compounds that regulate gene expression. Farnesol and tyrosol have been reported to have important function as signalling chemicals participating mainly in *C. albicans* regulatory mechanisms (Albuquerque and Casadevall 2012; Sebaa et al. 2019).

Tyrosol (2-(4-hydroxyphenyl)ethanol) is abundant in natural sources such as olive and has antioxidant properties. Most of the research on tyrosol has been conducted with *C. albicans* (Chen et al. 2004*),* but their role has also been investigated in other fungi (Wongsuk et al. 2016). In dimorphic fungi tyrosol can stimulate the morphological transition from spherical form to filamentous growth and promotes biofilm formation. Tyrosol has a greater effect on accelerating germ tube formation when farnesol is absent or limited in the environment (Nickerson et al. 2006). Whether tyrosol inhibits biofilm formation or stimulates germ tube formation depends on the applied concentration range (Alem et al. 2006; Cordeiro et al. 2015). The extent of inhibition also depends on the progress (stage) of the biofilm formation; tyrosol exerted a stronger action on biofilm formation than on preformed biofilms (Seeba et al. 2019).

Though tyrosol is well-known as a quorum sensing molecule in yeasts, its role in filamentous fungi belonging to the genus *Cunninghamella* (*C. elegans, C. blakesleeana and C. echinulata*) has also been studied. Khan et al. (2021) discovered that tyrosol exhibited a comparatively minor impact on the biofilm formation of *C. elegans* and *C. echinulata* and on the growth of these fungi on agar plates. However, when exogenous tyrosol was added to previously grown *C. blakesleeana* biofilm, biofilm degradation and detachment occurred, and new additional planktonic culture was observed, proving that these molecules specifically regulated biofilm growth in this species. Therefore, the effect of tyrosol is species-specific regarding its impact on biofilm growth. Furthermore*, Penicillium chrysogenum* DXY-1 also produces tyrosol, which has been found to inhibit the growth of bacterial biofilms (Chang et al. 2019).

Biofilm formation of *Candida albicans* has been widely studied and several practices were developed for its inhibition (Atriwal et al. 2021). The biofilm formation of *Candida boidinii* has not been studied although this species plays a decisive role in biotechnological processes and in the food industry.

One of the main objectives of this research was to assess the applicability of the high-throughput microtiter plate assay for quantification of biofilm formation of *Candida boidinii.* To improve the applicability of the method, numerous experimental parameters were varied to find the best adjustment for measuring quantitatively the biofilm formation capacity besides population growth in the cells. In addition, we aimed at extending our knowledge about the effect of different environmental parameters on the biofilm formation of *Candida boidinii*.

The further aim of this study was to test and evaluate the concentration- and time-dependent effect of tyrosol on the biofilm development of this yeast.

Our hypothesis was that tyrosol might act as a quorum-sensing molecule for biofilms as well as for planktonic cells in the case of *Candida boidinii* yeast similarly to the thoroughly studied *Candida albicans*. Modulation of the biofilm formation of *C. boidinii* with tyrosol and the systematic approach is unique in the scientific literature.

## Materials and methods

### Influence of different growth media, incubation time and incubation temperature on biofilm formation

#### Yeast strain and culture conditions

*Candida boidinii* strain ATCC 18810™ (from DSM, number 70026) was used in the research. The strain was maintained aerobically at 25 °C as agar slants culture on different yeast media; yeast extract-peptone-dextrose (YPD) agar, universal medium for yeasts (YM) agar and malt extract agar (Malt). For each experiment, 16 h old (overnight) shaken cell culture was prepared by inoculating 30 ml of the selected liquid growth media with one loopful of yeast colony.

#### Growth media composition and temperature assessment

The research was focused on the assessment of biofilm formation under different media compositions, incubation time and temperatures. The formation of biofilm was studied in three different media. Test growth media were YPD (10 g yeast extract l^−1^; 20 g bacteriological peptone l^−1^; 20 g glucose l^−1^), YM (3 g yeast extract l^−1^; 3 g malt extract l^−1^; 5 g peptone from soybeans l^−1^; 10 g glucose l^−1^) and Malt (20 g malt extract l^−1^; 1 g bacteriological peptone l^−1^; 5 g glucose l^−1^). The analysis of the effect of nutritional conditions was combined with three temperature settings (22 °C, 30 °C, and 37 °C). The measurements with all the possible variations of the selected factors were accomplished during the experiments.

#### Testing biofilm formation applying crystal violet staining

Evaluation of the applicability and further development of O'Toole (2011) method was aimed for determining the biofilm formation of *C. boidinii* yeast. To improve the reliability of the method, we varied some experimental settings to find the best setup for measuring biofilm formation and population growth in *C. boidinii*. Optimizing the cell density of the initial yeast inoculum is crucial for proper and stable formation of biofilm (Pierce et al. 2008) since QS-mediated mechanisms play a key role in the biofilm formation. Too high or too low cell density may result in poor biofilm. The cell density of the initial inoculum was optimized in preliminary studies for experiments conducted with 96-well microtiter plates. Briefly, the overnight culture was spectrophotometrically set to reach a final absorbance of 0.2 at 600 nm. A total of 200 μl of suspension was inoculated to the selected wells in 96-well trays and incubated for different periods of time (24, 48, 72, and 96 h) at the selected temperature. The measurements with all the possible variations of the selected factors were accomplished during the experiments. The experiments were carried out in 96-well round-bottomed sterile polystyrene microplates. After incubation, the growth medium was decanted, and the biofilm was gently washed two times by submerging the plate in a tub filled with water in order to remove nonadherent cells. Then 250 μl of a 0.1% aqueous crystal violet (CV) solution was added to each well and plates were incubated for 15 min to stain the cells. Then the excess solution was removed, and the washing steps were repeated carefully not to damage the biofilm inside the wells. To solubilize the biofilm-bound CV 250 μl of a 30% acetic acid solution was added to each well. After 15 min exposure time 250 μl of the extract was transferred to a new 96-well plate. The absorbance was measured spectrophotometrically at 544 nm with Fluostar Optima BMG Labtech microplate reader. All steps were carried out at room temperature. This method enables the measuring of cells attached both to the bottom as well to the walls of the wells of 96- well plates.

To increase the sensitivity of the method a fixing step with methanol was included based on Peeters et al. (2008). 250 μl of 99% methanol was pipetted in the wells after the decantation of the growth medium and the first washing steps. After 15 min the methanol was removed, and the plates were completely dried under laminar flow.

#### Optical density measurement testing cell density

To investigate the potential toxic effect of tyrosol via measuring the growth of the yeast population, an optical density (OD) assay was performed as described by Molnár et al. (2021). The optical density of the wells was recorded before and after the incubation. To monitor the planktonic cells 150 µl supernatant from the wells was transferred to a new microplate, carefully not to damage the biofilm layers and optical density was measured again. The optical density was measured at a wavelength of 630 nm with a microplate reader (DIALAB ELx800 ELISA Microplate Reader).

### Testing the effect of tyrosol on *Candida boidinii*

To evaluate the effect of tyrosol on the cell density in cultivation, morphology, and biofilm formation of *C. boidinii* in YPD, 50 μl of various tyrosol (Sigma) concentrations ranging from 6.25 mM to 100 mM dissolved in distilled water were added to the wells of 96-well microtiter plates. Also, 50 μl of distilled water was used as a negative control. Then 150 μl of the previously mentioned overnight culture suspension was added to the wells. The final volume in each well was 200 μl. Then, the microplates were incubated and treated as described above.

The biofilm formation was tested by crystal violet staining (as described above) and XTT assay for testing viability of the biofilm as well.

Morphological alterations were monitored using a Nikon Eclipse E400 microscope under 600 × magnification and representative images were captured using SPOT imaging software (Diagnostic Instruments).

#### Testing biofilm viability applying XTT assay

A tetrazolium reduction assay with the 2,3-bis(2-methoxy-4-nitro-5-sulfophenyl)-5-[(phenylamino) carbonyl]-2H-tetrazolium hydroxide (XTT) was used as an additional measurement to obtain a more detailed picture of the biofilm formation (e.g. biofilm metabolic activity). The metabolic activities of living cells within biofilms were characterized by using XTT-reduction assay applying the same experimental setup as in the case of the biofilm formation test applying crystal violet staining. The XTT colorimetry assay was performed according to Jin et al. (2003) with some modifications. Prior to each assay the sodium salt of XTT (Sigma) was dissolved in saline at 1 mg XTT ml^−1^ and Menadione (Sigma) was dissolved in acetone to give a concentration of 0.4 mM. The solutions were filter sterilized through a 0.22-μm-pore-size filter. Then the XTT/Menadione reagent was prepared that contained 5 parts XTT/1 part Menadione. After incubation, the biofilms were washed five times with 250 μl of PBS, and then 250 μl of PBS and 15 μl of the XTT/Menadione solution was added to each of the wells. The microtiter plate was then incubated in the dark for 2 h at 37 °C. Following incubation, 100 μl of solution was transferred to new wells. A colorimetric change in the XTT-reduction assay was measured in a microtiter plate reader (DIALAB ELx800 ELISA Microplate Reader) at 450 nm.

### Statistical analysis

All the experiments were done in five replicates and standard deviation from the mean was calculated. The effect of experimental parameters on biofilm formation was analysed using Repeated Measures ANOVA (RMANOVA) by TIBCO Statistica™ 13.5 (TIBCO Software, Inc., Palo Alto, CA, USA) software. To verify the criteria Mauchley sphericity test was applied. Statistical analysis were performed at the p < 0.05 significance level. In the figures, significant effects are indicated by letters in alphabetical order. Columns signed with the same letter indicate that there was no significant difference between them.

## Results

### Influence of different growth media, incubation time and incubation temperature on biofilm formation

As part of method development, we investigated the effect of the medium, incubation temperature, and incubation time on the growth and biofilm formation of *Candida boidinii* (Table 1). Based on the RMANOVA analysis, both the microbial growth and biofilm formation were significantly affected by the growth media, incubation time, and incubation temperature. Their interactions also had a significant effect on the growth and biofilm development of *C. boidinii* in each combination.

**Table 1:** RMANOVA results over time to evaluate the effects of medium, and incubation temperature on the growth and biofilm formation of *C. boidinii*, bold numbers (MS, F) indicate significant differences at p < 0.05

	Growth				Biofilm formation		
Source of variation	d.f	MS	F	*P*	MS	F	*p*
Growth media	2	**62.39**	**578.3**	0.00	**0.42**	**255.4**	0.00
Temperature	2	**258.33**	**2394.4**	0.00	**1.96**	**1206.0**	0.00
Time	3	**5.86**	**129.2**	0.00	**0.61**	**252.0**	0.00
Growth media × Temperature	4	**3.02**	**28.0**	0.00	**0.16**	**98.4**	0.00
Time × Growth media	6	**0.32**	**7.0**	0.00	**0.04**	**17.5**	0.00
Time × Temperature	6	**0.85**	**18.7**	0.00	**0.11**	**46.1**	0.00
Time × Growth media × Temperature	12	**0.500**	**10.98**	0.00	**0.0505**	**21.0**	0.00

*d.f* Degree of freedom, *MS* mean square, *F* F-ratio, *p* *p*-value

Relative (growth-related) biofilm formation was also significantly affected by the growth media, incubation time and temperature (Supplementary Table 1, Supplementary Fig. 1).

**Fig. 1:**
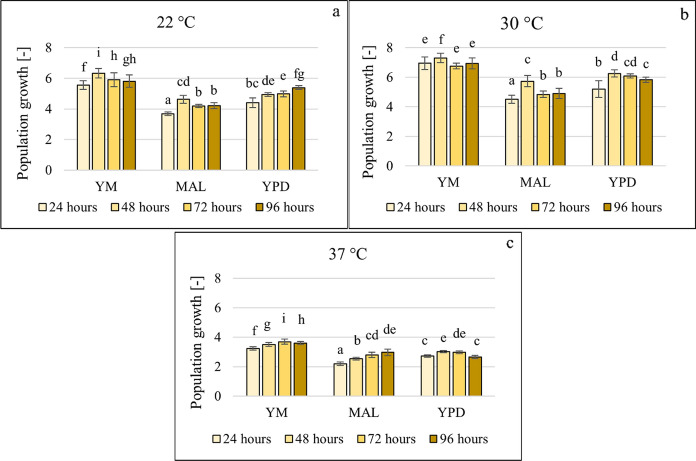
Growth of *Candida boidinii* in three different media at 22 °C (**a**), 30 °C (**b**) and 37 °C (**c**), data represent averages of five replicates

**Fig. 2:**
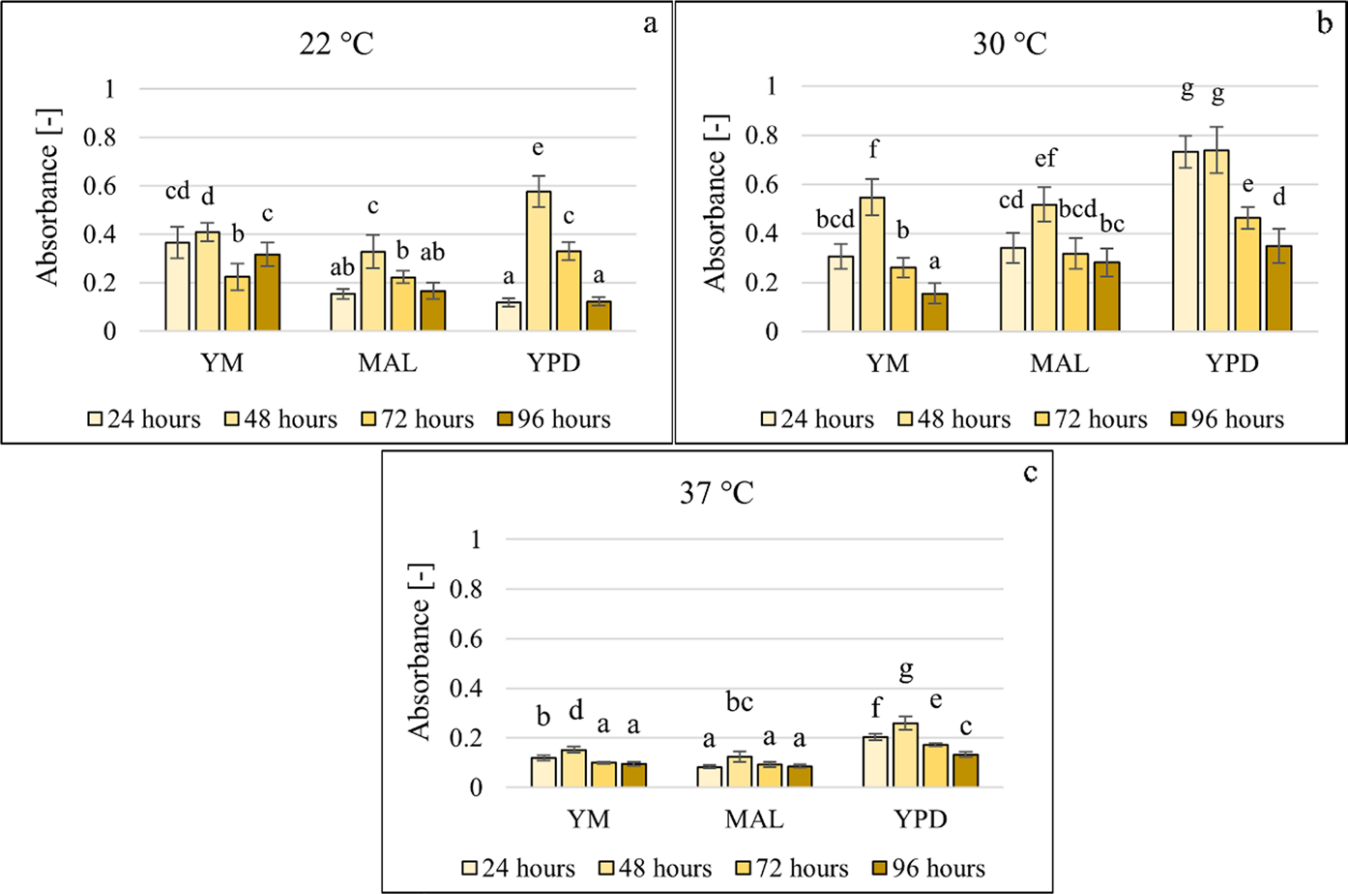
Biofilm formation of *Candida boidinii* in three different media at 22 °C (**a**), 30 °C (**b**) and 37 °C (**c**), data represent averages of five replicates

All three media were suitable for the growth of the yeast, but small differences were observed (Fig. 1). Concerning temperature, the highest biofilm formation was found at 22–30 °C, and 37 °C was the least favourable for both growth and biofilm formation (Fig. 1 and Fig. 2). Population growth was calculated as the ratio of the optical density values measured after the specific incubation time (24, 48, 72 and 96 h) and the start time at 630 nm.

37 °C (**c**), data represent averages of five replicates.

After 24 h, *C. boidinii* formed biofilms in all three media at 22 °C and 30 °C. The effect of incubation time is most evident at 22 °C in YPD. A build-up phase is observed after 24 and 48 h, followed by a degradation phase. The degradation phase can be observed after 72 h in almost all combinations; therefore, testing the effect of additives after 72 h may not be relevant.

### Effect of tyrosol on growth, biofilm formation and viability

**Fig. 3:**
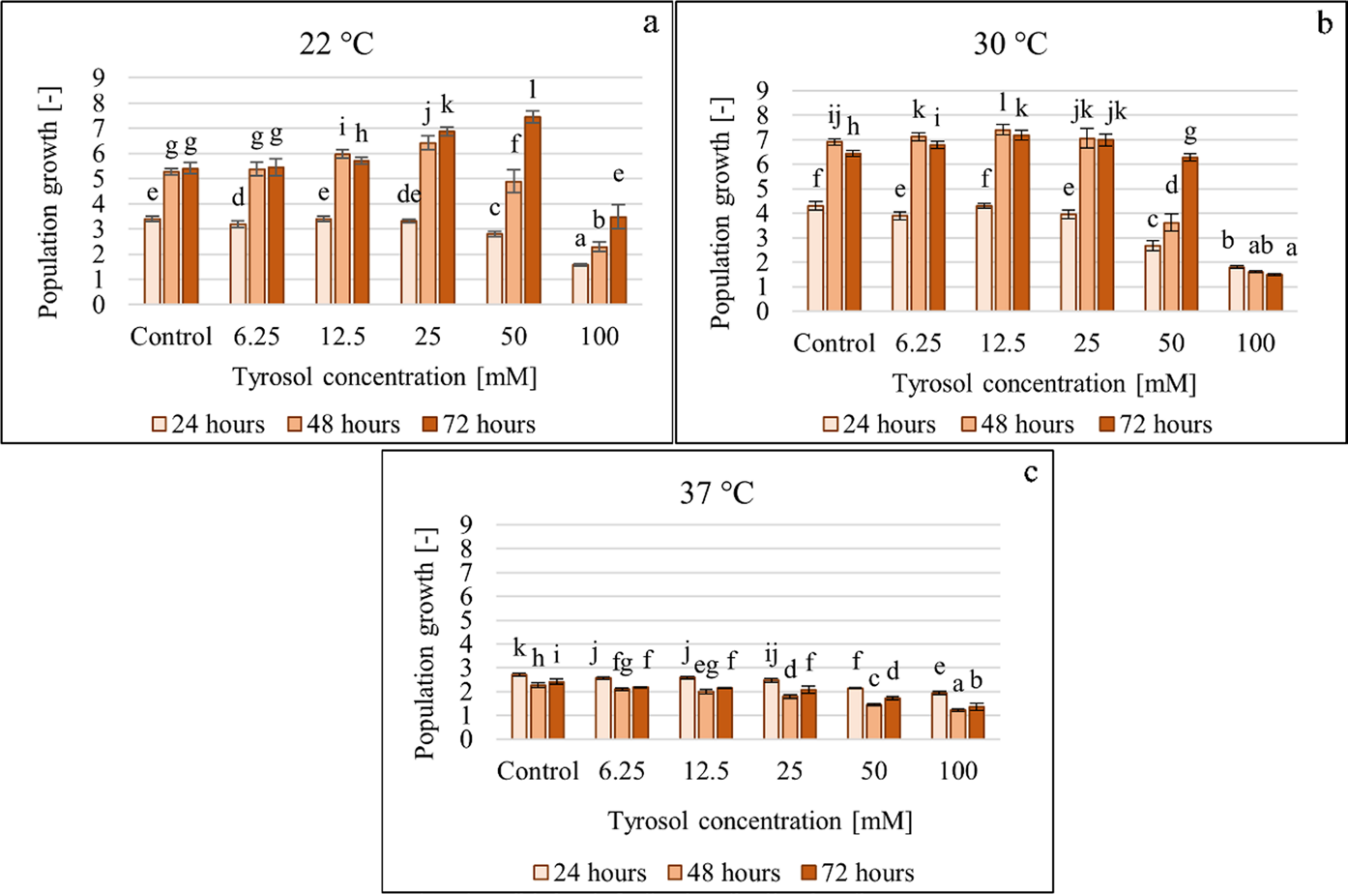
Effect of increasing tyrosol concentration on microbial growth at 22 °C (**a**), 30 °C (**b**) and 37 °C (**c**), data represent averages of five replicates

**Fig. 4:**
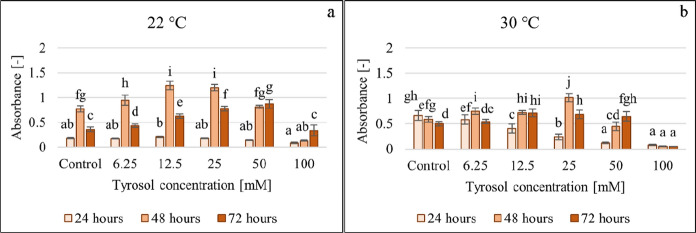
Effect of increasing tyrosol concentration on CV-stained biofilm formation at 22 °C (**a**) and 30 °C (**b**), data represent averages of five replicates

The effect of tyrosol on the microbial growth of *C. boidinii* in YPD was tested within 6.25–100 mM concentration in 96-well microtiter plates studying also the effect of the incubation temperature and contact time. Population growth (Fig. 3) was determined based on the increase in optical density (OD) relative to baseline in each cell of the microtiter plate. The developed and crystal violet stained biofilm (Fig. 4) was quantified and characterized based on the absorbance measured at 544 nm.

The RMANOVA analysis (Table 2, Supplementary Table 2) demonstrated that both yeast growth and biofilm formation were significantly affected by the tyrosol concentration, incubation time and temperature. Their interactions also had a significant effect on microbial growth in every combination.

**Table 2:** RMANOVA results over time and temperature to evaluate the effects of tyrosol on the growth and CV-stained biofilm formation of *C. boidinii*, bold numbers (MS, F) indicate significant differences at p < 0.05

	Growth				Biofilm formation (CV-stained)		
Source of variation	d.f	MS	F	p	MS	F	p
Tyrosol	5	**59.46**	**2496.5**	0.00	**0.97**	**543.6**	0.00
Temperature	2	**319.30**	**13,364.2**	0.00	**6.79**	**3791.9**	0.00
Time	2	**83.92**	**2472.8**	0.00	**2.40**	**1110.2**	0.00
Tyrosol × Temperature	10	**13.32**	**559.1**	0.00	**0.30**	**166.7**	0.00
Time × Tyrosol	10	**5.32**	**156.8**	0.00	**0.26**	**120.5**	0.00
Time × Temperature	4	**39.12**	**1152.7**	0.00	**1.09**	**502.3**	0.00
Time × Tyrosol × Temperature	20	**1.90**	**55.9**	0.00	**0.10**	**47.6**	0.00

*d.f* Degree of freedom, *MS* mean square, *F* F-ratio, *p* *p*-value

The effects of different tyrosol concentrations were time-, temperature-, and concentration-dependent (Fig. 3).

At 22 °C incubation temperature after 24 and 48 h, tyrosol inhibited population growth (total cell density) by 7–56% in the 50–100 mM concentration range. However, the inhibition of 50 mM tyrosol turned into 37% stimulation after 72 h. This increase in growth was also observed after 48 h (14–22%) in the 12.5–25 mM tyrosol concentration range, which stimulation persisted after 72 h (5–27%). 100 mM tyrosol concentration inhibited growth up to 56% at all three incubation times. At 30 °C 3–77% inhibition was observed in the 50–100 mM range after 24, 48, and 72 h.

The results demonstrated that the 37 °C incubation temperature was the least favourable for the growth of *C. boidinii*, while the highest level of biofilm development (characterized by CV-staining) was observed at 30 °C.

Figure 4 illustrates the effect of tyrosol on CV-stained biofilm formation determined at 22 °C and 30 °C.

Similarly to the results on microbial growth of *C. boidinii,* the results on its biofilm formation (determined by CV-staining) at 37 °C were also unfavourable (Supplementary Fig. 2).

**Fig. 5:**
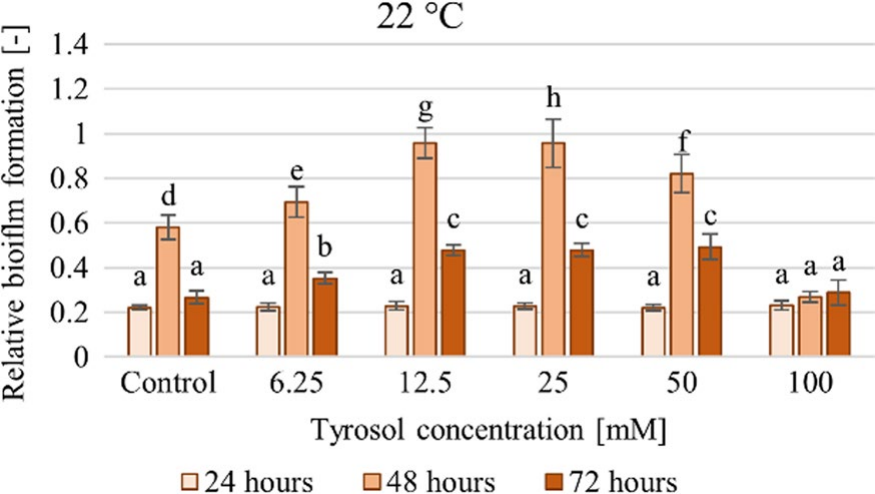
Effect of increasing tyrosol concentration on relative biofilm formation at 22 °C, data represent averages of five replicates

Tyrosol differentially affected yeast growth and biofilm formation. Tyrosol had different effects after 24 h of incubation at the two temperatures. After 24 h at 22 °C the additive had no significant effect, but, at a higher temperature a concentration-dependent inhibition of the biofilm formation was observed. At 22 °C the longer the incubation lasted, the higher was the inhibitory concentration. After 48 h, tyrosol at up to 25 mM concentration, stimulated biofilm formation (23–61%) in a concentration-dependent manner. After 72 h, this effect occurred at up to 50 mM (21–143%). This concentration-dependent stimulation was less noticeable at 30 °C. The results also point to the fact that tyrosol at 100 mM concentration resulted high antifungal effect, therefore it inhibited both growth and biofilm formation. Relative (growth-related) biofilm formation was determined by dividing the CV-stained biofilm formation data measured at 544 nm by the optical density measured at 630 nm (for the given treatment). Relative (growth-related) biofilm formation corroborates the effect of tyrosol at 22 °C (Fig. 5). At up to 50 mM concentration tyrosol had no or a single stimulatory effect (at all contact times), while at a concentration of 100 mM a clear inhibition was observed.

The effect of increasing tyrosol concentrations on relative biofilm formation at 30 °C and 37 °C can be found in the supplementary materials (Supplementary Fig. 3). Different The results obtained at 30° C are different from those experienced at 22 °C. Tyrosol exhibited a concentration-dependent inhibition (3–17%) after 24 h, but after 48 and 72 h stimulatory effects were also observed at lower concentrations (6.25–50 mM).

**Fig. 6:**
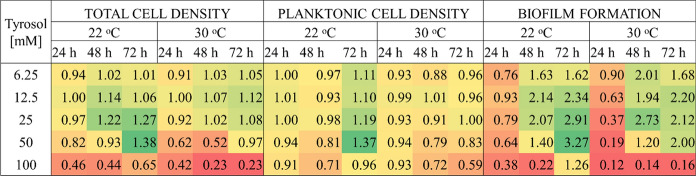
Summary of tyrosol concentration-, contact time- and temperature-dependent effects on the growth (total and planktonic cell density) and the biofilm formation of *C. boidinii*

Figure 6 summarizes the concentration-, temperature and time-dependent effect of tyrosol on total and planktonic cell density as well as biofilm formation illustrating that 2-(4-hydroxyphenyl)ethanol differentially affected *Candida boidinii* growth and biofilm formation.

After 24 h at both 22 °C and 30 °C, the tyrosol added at time zero was able to reduce strongly the biofilm formation, while it had little effect on the growth of planktonic cells. However, after 48 and 72 h, although there were differences between the data obtained at 22 °C and 30 °C, only 100 mM tyrosol reduced the biofilm formation; and the 6.25–50 mM concentrations clearly exhibited stimulating effects on biofilm formation.

**Fig. 7:**
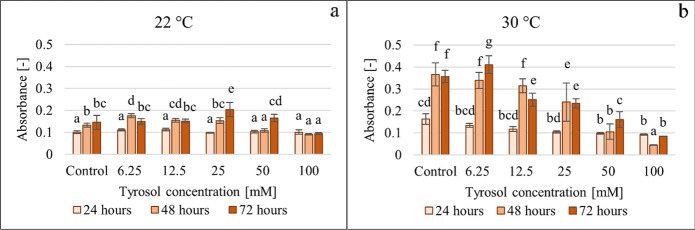
Effect of increasing tyrosol concentration on the biofilm metabolic activity determined by XTT-assay at 22 °C (**a**) and 30 °C (**b**), data represent averages of five replicates

Figure 7 shows the effect of tyrosol on the metabolic activity of the formed biofilm determined at 22 °C and 30 °C applying XTT-assay.

The biofilm formation assay based on metabolic activity reflected more sensitively the differences in the extent of biofilm formation in terms of temperature, tyrosol concentration, and exposure time. The RMANOVA analysis (Supplementary Table 3) demonstrated that biofilm metabolic activity was significantly influenced by the tyrosol concentration, incubation time and temperature. Their interactions also had a significant effect on biofilm formation capacity in every combination.

At 22 °C incubation temperature after 24 h, none of the tyrosol concentrations had a significant effect. After 48 h at 22 °C the tyrosol had significant effect on the metabolic activity of the biofilm; significant increase compared to control was observed at 6.25 and 12.5 mM concentration (~ 32% and ~ 17% increase, respectively), while at 50 and 100 mM tyrosol addition the biofilm metabolic activity was inhibited (~ 19% and 31%, inhibition, respectively).

Meanwhile, at 30 °C after 24 h, tyrosol decreased the biofilm metabolic activity proportionally with the concentration, which became already significant at 50 and 100 mM, resulting in a ~ 40% and ~ 43% decrease, respectively.

After 72 h at 22 °C, tyrosol at up to 50 mM concentration, stimulated biofilm formation (2–40%), 100 mM tyrosol resulted in ~ 36% decrease in the biofilm formation determined by biofilm metabolic activity.

Based on the measurement of biofilm metabolic activity at 30 °C incubation temperature, the reductions in biofilm formation were much greater than at 22 °C. After 48 h the inhibition ranging on average between 7 and 88% was proportional to the tyrosol concentration considering the entire tested concentration range (6.25–100 mM). After 72 h, the lowest tyrosol concentration had a stimulating effect on the metabolic activity, while in the 12.5–100 mM concentration range, it showed an inhibitory effect (29–76%) proportional to the concentration.

These results point out that tyrosol exhibits a high inhibitory effect on metabolic activity at 30 °C at high (> 12.5) concentration levels.

## Discussion

### Evaluation of the experimental parameters on the growth and biofilm formation of *Candida boidinii*

Biofilm development is a process dependent on several factors (e.g. available nutrients, temperature, pH, solid surface), and the factors should be treated as an integrated system in which each variable interacts to promote the development of an extracellular polymeric matrix and the adhesion of the cells. Based on the examination of the effects of the incubation time, the biofilm mass increased after the first 2 days of incubation, but usually, this was followed by biofilm degradation.

Although there are several techniques for the assessment of the biofilm formation of *Candida* and other yeast species (Ramage et al. 2001; Pierce et al. 2008; Speranza et al. 2020), many researchers found the 96-well microtiter plate method to be the most useful applying various endpoint measurement methods.

The original methodology used in this research was a microtiter plate assay applying crystal violet staining of the biofilm established by O’Toole’ (O'Toole 2011). However, we have extended the duration of the method to study the formation and degradation phases of the biofilm, as well as to assess the effect of various experimental settings in the different stages of biofilm development. Biofilm formation and degradation differed over time, temperature, and growth media. Regarding the incubation time, the most considerable amount of biofilm was formed after 48 h at all temperatures. At longer incubation times, the degradation of the biofilm was visible. Since there are no examples of such studies in the literature with *C. boidinii*, this research fills a gap. While in the most related research with other yeasts using this method (Ramage et al. 2001; Pierce et al. 2008; Speranza et al. 2020), only 24 or 48-h contact time measurements were applied, in our research, we observed that even after 72 h a stable biofilm was formed in the YPD culture medium at 22 and 30 °C. Therefore, based on this, the 3-day incubation period is recommended.

As previous research has already shown, the maximum temperature for the growth of *C. boidinii* is 36.5 °C while the optimum is about 27 °C (Romero-Gil et al. 2013). Our results confirmed that the incubation temperature of 37 °C was unfavourable for this microorganism both in terms of growth and biofilm formation. But, both 22 °C and 30 °C incubation temperatures may be relevant in biotechnological applications.

The effect of medium composition as a potential influencing factor on growth and biofilm formation was also studied in our research since the nutritional supply plays a crucial role in growth and biofilm formation.

Generally, the growth media for fungi contain carbon and nitrogen sources. In our study, all the media used were general-purpose media for the cultivation of yeast, which differed primarily in the amount of glucose and nitrogen source (peptone). Due to this, based on RMANOVA analysis, our study showed statistically significant differences both in the growth and biofilm formation function of the media composition. The YPD medium contained the highest concentration of glucose and peptone, while the malt extract medium contained the least. The YM medium represented a medium level of these nutrients among the tested media.

In terms of population growth, the YM medium was the most favourable at all temperatures, and the smallest growth occurred in the malt extract media in all cases. Regarding biofilm formation, the nutrient-rich YPD media were the most favourable in promoting the highest biofilm formation, in accordance with studies performed by *Candida* and *Saccharomyces* species. Speranza et al. found that the biofilm formation of *S. cerevisiae* was more pronounced in nutrient-rich growth media (Speranza et al. 2020). Non-albicans *Candida* species also presented a greater amount of biofilm formation in YPD media than in the synthetically defined RPMI 1640 media, indicating that the richer the medium, the greater the biofilm formation (Tan et al. 2016).

In summary, the temperature and growth media have an impact on biofilm development and need pre-evaluation for the specific biotechnological application of the *C. boidinii*.

### Effect of tyrosol on the growth and biofilm formation of *Candida boidinii*

The use and exploitation of microbial QS mechanisms for biotechnological purposes is a developing field in bacteriology, but there are fewer examples in the field of fungi, despite many yeasts having biotechnological applications. Nevertheless, influencing biofilm formation in yeasts has recently gained attention due to the infectiousness of some species and their biotechnological application. The knowledge of *C. boidinii* biofilm formation ability and its influencing factors is of considerable economic importance for food-processing companies. For example, a greater tendency to form biofilm may be advantageous in biomass separation in fermentation processes. Thus, the investigation of potential signal molecules of QS processes is increasingly important.

Since tyrosol—as a quorum-sensing molecule in yeasts—has well-known effects on many yeasts and was reported to reduce lag phase and induce hyphae formation (Nath et al. 2021), one of our main goals was to study the effect it had on the growth and biofilm formation of *C. boidinii* as a function of time, concentration, and temperature. Its effect has not been studied at all in this yeast, even though it can also affect the fermentative capacity of this *Candida* species, which can provide useful information on its use in various biotechnological processes.

Tyrosol is reported as stable compound, it is more resistant to autoxidation than other polyphenols, and retains its antioxidant activity even under less favourable conditions (Karković Marković et al. 2019). Tyrosol also demonstrated antimicrobial activity (*E. coli*) through binding to and inhibiting bacterial ATP synthase (Amini et al. 2017).

Chen et al. (2004) proved that the human fungal pathogen *Candida albicans* exhibited a significant delay in growth (longer lag phase) in the absence of tyrosol diluting an overnight culture of the organism into fresh minimal medium. However, the lag phase extremely shortened after adding to the *C. albicans* cells cell-free supernatant (containing tyrosol) from high-density cultures at low densities. They also proved the tyrosol-mediated dynamics of both growth and morphogenesis, since tyrosol accelerated the morphological conversion of yeast-form cells to filaments in this yeast.

Alem et al. (2006) investigated the relationship between tyrosol level and biofilm formation in *C. albicans* strains. The cells of both the planktonic phase and the biofilm secreted tyrosol, and there was a correlation between extracellular tyrosol production and biomass for both cell types. However, biofilm cells secreted at least 50% more tyrosol (relative to dry biomass) than planktonic phase cells.

Exogenous tyrosol (at final concentrations between 20 μM and 1 mM) appeared to have no effect when determining biofilm formation based on quantitative tetrazolium reduction but scanning electron microscopy showed that tyrosol stimulated hyphal production in the early stages of biofilm development (1–6 h).

During our research so far, we did not observe morphological changes in the *C. boidinii* yeast under the influence of tyrosol in the tested concentration range and with the used experimental settings similarly to the Jakab et al.’s (2019) research, but at the same time, it affected both growth and biofilm formation depending on the concentration, contact time and the temperature. As shown in the Supplementary Fig. 4, there was no transformation from yeast to filamentous form at any concentration. This confirms that the mechanism of action of tyrosol is species-specific regarding its effect on morphology similarly to Khan et al.’s (2021) research.

Regarding the tyrosol-mediated effect on growth and biofilm formation, these endpoints were found to be both stimulated and inhibited depending on the concentration, contact time and temperature.

At the highest tested concentration (100 mM), tyrosol clearly inhibited both growth and biofilm formation at all incubation temperatures and contact times. This inhibition occurring at high concentrations clearly indicates a potential antifungal and cytotoxic effect; tyrosol used in higher concentrations has been shown to be toxic to certain microorganisms in several studies at supraphysiological concentration (Kovács and Majoros 2020).

At the same time, the exogenous tyrosol added to the system at lower concentrations (6.25–50 mM) affected these endpoints to different extent depending on the temperature, contact time and end point, clearly illustrating that its effect in the complex system was influenced by numerous parameters.

As reported by Sebaa et al. (2019), 1–20 mM tyrosol inhibited the development of *Candida albicans* biofilm. They found that tyrosol exerted a stronger action on biofilm formation than on preformed biofilms. Cordeiro et al. (2015) reported similar results, showing that high concentrations of tyrosol (2.5 and 5 mM) alone or in combination with itraconazole, and fluconazole promoted significant reductions in biofilms formed by several strains of *C. albicans* and *C. tropicalis*. The exogenous tyrosol may interfere with ergosterol biosynthesis causing the reduction of biofilm formation in the first 24 h as reported by Cordeiro et al. (2015). Comparing our research outcomes with these literature results, we could find differences in the extent of the tyrosol-mediated effect on biofilm formation. While inhibitory effects have been experienced even in the 1–20 mM concentration range based on some studies (Cordeido et al. 2015; Sebaa et al. 2019), our results demonstrated stimulation or only slight inhibition at this concentration range. This can obviously be related to the different yeast strains, the different composition of the culture media, but most of all to the different contact times.

There is a lack of studies in the literature about the stimulating effect of tyrosol on biofilm formation, which clearly illustrates that the biological effects of tyrosol on yeasts is not well understood and justifies the need for long-term (more than 24 h) studies to assess the biofilm formation at different tyrosol concentrations. Our results also suggest that the tyrosol added at time zero was biotransformed by the cells over time.

For initial assessment of the biofilm development capability of a yeast, a rapid, cost-effective, reliable biofilm characterization methodology is an essential step. Several different methods have been developed for testing biofilm formation capacity providing deeper knowledge on biofilm physiology, structure, and composition (Azeredo et al. 2017). Despite the numerous methodological developments, there is a lack of consensus among the diverse techniques used in determination of biofilm formation.

Although the high-throughput microtiter plate based crystal violet staining method is one of the most widely used techniques among these methods to estimate biofilm formation, it also has disadvantages. The main disadvantage is that it also stains dead cells, so it does not provide information about the viability, physiology of the cells in the biofilm.

To eliminate this limitation, we also performed comparative test, e.g. microtiter plate based XTT-reduction assay, to assess the effect of tyrosol on biofilm formation. XTT-reduction assay is an indirect measurement of biofilm metabolic activity by chemical reduction of the applied tetrazolium salt. Both crystal violet staining and the XTT-reduction assay reflecting the metabolic activity were used with the same settings to characterize the degree of biofilm formation. Although this method is much more expensive and more time-consuming due to the extra steps during execution, its sensitivity exceeded that of the crystal violet staining method.

In our research, the results of the XTT-reduction assay based on the metabolic activity test were generally consistent (at 22 and 30 °C) with the results of the growth tests, clearly illustrating that this method reflects better the viability of the cells in the biofilm than the crystal violet staining. Based on our results, the crystal violet staining method can be used as a first screening or preliminary testing due to its time- and cost-efficiency to assess the effect of experimental parameters or additives affecting biofilm formation. The more expensive and sensitive XTT-assay is recommended, for example, to characterize the fine, concentration-dependent effects since it allows differentiating between total and dead cells.

One of the main significances of this study lies in extending our knowledge about the effect of different environmental conditions on the growth and biofilm formation of the *Candida boidinii* strain. A better understanding of these traits could be an important tool in influencing both the QS-regulated processes and the efficiency of the fermentation processes carried out with *C. boidinii.*

Our results support the assumption that tyrosol may act as a quorum-sensing molecule for biofilm development by *C. boidinii* as well, and its effect is significant both in the early and intermediate stages of biofilm formation.

## Conclusion

In conclusion, this research clearly presented the biofilm-forming ability of *C. boidinii* and demonstrated that the biofilm formation is affected by the growth media and temperature. The applicability of the microtiter plate assay as a high-throughput screening tool for characterizing the influence of tyrosol on the growth and biofilm formation capacity of *C. boidinii* was also demonstrated. The exogenous tyrosol exerted concentration-, temperature and time-dependent effects on the population growth and biofilm formation ability of *C. boidinii* yeast. This is the first report about the influencing capacity of tyrosol on the growth and biofilm formation of *C. boidinii*, where the molecule was found to show both decreasing and enhancing effects depending on its concentration, contact time and temperature. Our results indicate that tyrosol may be a signalling molecule in this *Candida* species as well and predict its potential application as a regulating factor of various fermentations by this yeast.

### Supplementary Information

Below is the link to the electronic supplementary material.

Supplementary file1 (DOCX 1537 KB)

## Data Availability

Experimental data are available within this research article and in the related Supplementary Materials. The raw datasets generated during and/or analysed during the current study are available from the corresponding author on reasonable request.
