# Supplementary material for: Biofilm development of Candida boidinii and the effect of tyrosol on biofilm formation
Source: Biotechnol Lett. 2023 Oct 13;45(11-12):1541–54. doi: 10.1007/s10529-023-03432-5 (PMC10635961; doi:10.1007/s10529-023-03432-5)
Supplement: Supplementary file 1 — Supplementary file1 (DOCX 1537 KB) [file 10529_2023_3432_MOESM1_ESM.docx]

**SUPPLEMENTARY MATERIALS**

**Biotechnology Letters** **(BILE-D-22-00684 revised)**

Biofilm development of *Candida boidinii* and the effect of tyrosol on biofilm formation

Rita Márton, Blanka Nagy, Mónika Molnár*

Budapest University of Technology and Economics Department of Applied Biotechnology and Food Science, Műegyetem rkp. 3. H-1111 Budapest, Hungary

*Corresponding author: [molnar.monika@vbk.bme.hu](mailto:molnar.monika@vbk.bme.hu)

**Supplementary Table 1** RMANOVA results over time to evaluate the effects of medium, and incubation temperature on the relative biofilm formation of *Candida boidinii,* bold numbers (MS, F) indicate significant differences at p < 0.05

| Source of variation | d.f | MS | F | *p* |
| --- | --- | --- | --- | --- |
| Growth media | 2 | **0.0219** | **12.9** | 0.00 |
| Temperature | 2 | **0.3350** | **197.1** | 0.00 |
| Time | 3 | **0.3951** | **255.9** | 0.00 |
| Growth media x Temperature | 4 | **0.0885** | **52.1** | 0.00 |
| Time x Growth media | 6 | **0.0206** | **13.3** | 0.00 |
| Time x Temperature | 6 | **0.0356** | **23.1** | 0.00 |
| Time x Growth media x Temperature | 12 | **0.0390** | **25.24** | 0.00 |

d.f: Degree of freedom; MS: mean square; F: F-ratio; *p*: *p*-value.


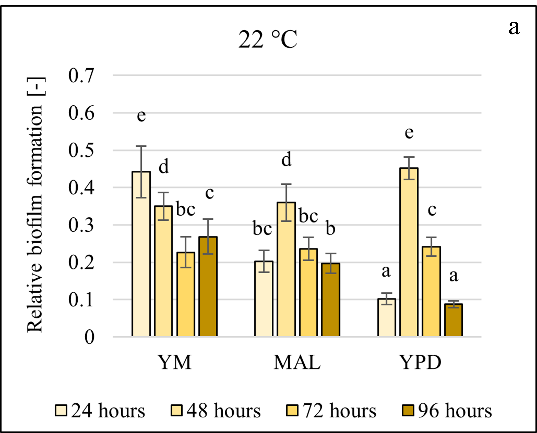

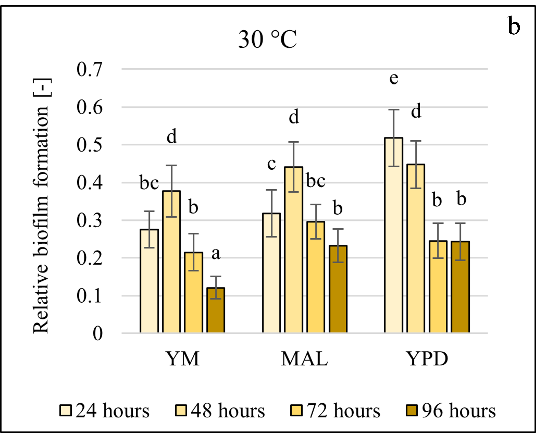


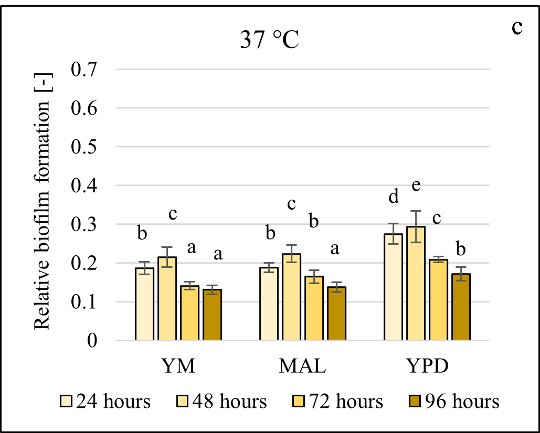


**Supplementary Fig. 1** Relative biofilm formation of *Candida boidinii* in three different media at 22 °C (a), 30 °C (b) and 37 °C (b), data represent averages of five replicates


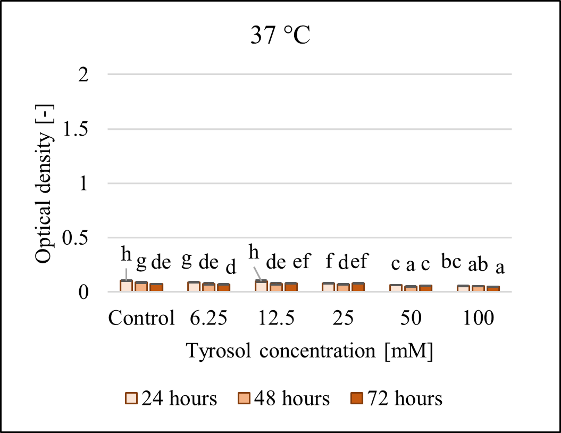


**Supplementary Fig. 2** Effect of increasing tyrosol concentration on CV-stained biofilm formation at 37 °C, data represent averages of five replicates


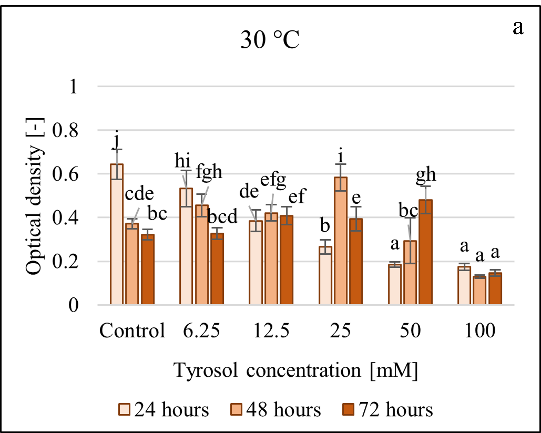

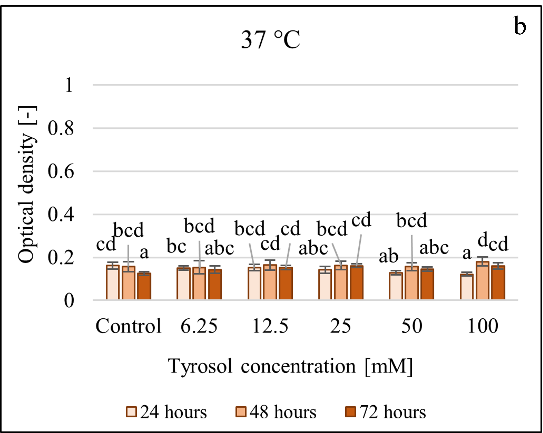


**Supplementary Fig. 3** Effect of increasing tyrosol concentration on relative biofilm formation at 30 °C (a) and 37 °C (b), data represent averages of five replicates

**Supplementary Table 2** RMANOVA results over time and temperature to evaluate the effects of tyrosol on the growth of planktonic cells of *C. boidinii*, bold numbers (MS, F) indicate significant differences at p < 0.05

| Source of variation | d.f | MS | F | p |
| --- | --- | --- | --- | --- |
| Tyrosol | 5 | **0.0106** | **78.7** | 0.00 |
| Temperature | 2 | **0.0510** | **377.8** | 0.00 |
| Time | 2 | **0.0362** | **305.3** | 0.00 |
| Tyrosol x Temperature | 10 | **0.0028** | **20.6** | 0.00 |
| Time x Tyrosol | 10 | **0.0021** | **17.7** | 0.00 |
| Time x Temperature | 4 | **0.0143** | **120.6** | 0.00 |
| Time x Tyrosol x Temperature | 20 | **0.0019** | **16.3** | 0.00 |

d.f: Degree of freedom; MS: mean square; F: F-ratio; *p*: *p*-value.

**Supplementary Table 3** RMANOVA results over time and temperature to evaluate the effects of tyrosol on the biofilm formation measured with the XTT assay of *C. boidinii*, bold numbers (MS, F) indicate significant differences at p < 0.05

| Source of variation | d.f | MS | F | p |
| --- | --- | --- | --- | --- |
| Tyrosol | 5 | **0.1180** | **198.5** | 0.00 |
| Temperature | 1 | **0.2586** | **435.1** | 0.00 |
| Time | 2 | **0.1559** | **259.5** | 0.00 |
| Tyrosol x Temperature | 5 | **0.0678** | **114.1** | 0.00 |
| Time x Tyrosol | 10 | **0.0196** | **32.6** | 0.00 |
| Time x Temperature | 2 | **0.0394** | **65.5** | 0.00 |
| Time x Tyrosol x Temperature | 10 | **0.0109** | **18.1** | 0.00 |

d.f: Degree of freedom; MS: mean square; F: F-ratio; p: p-value.

**
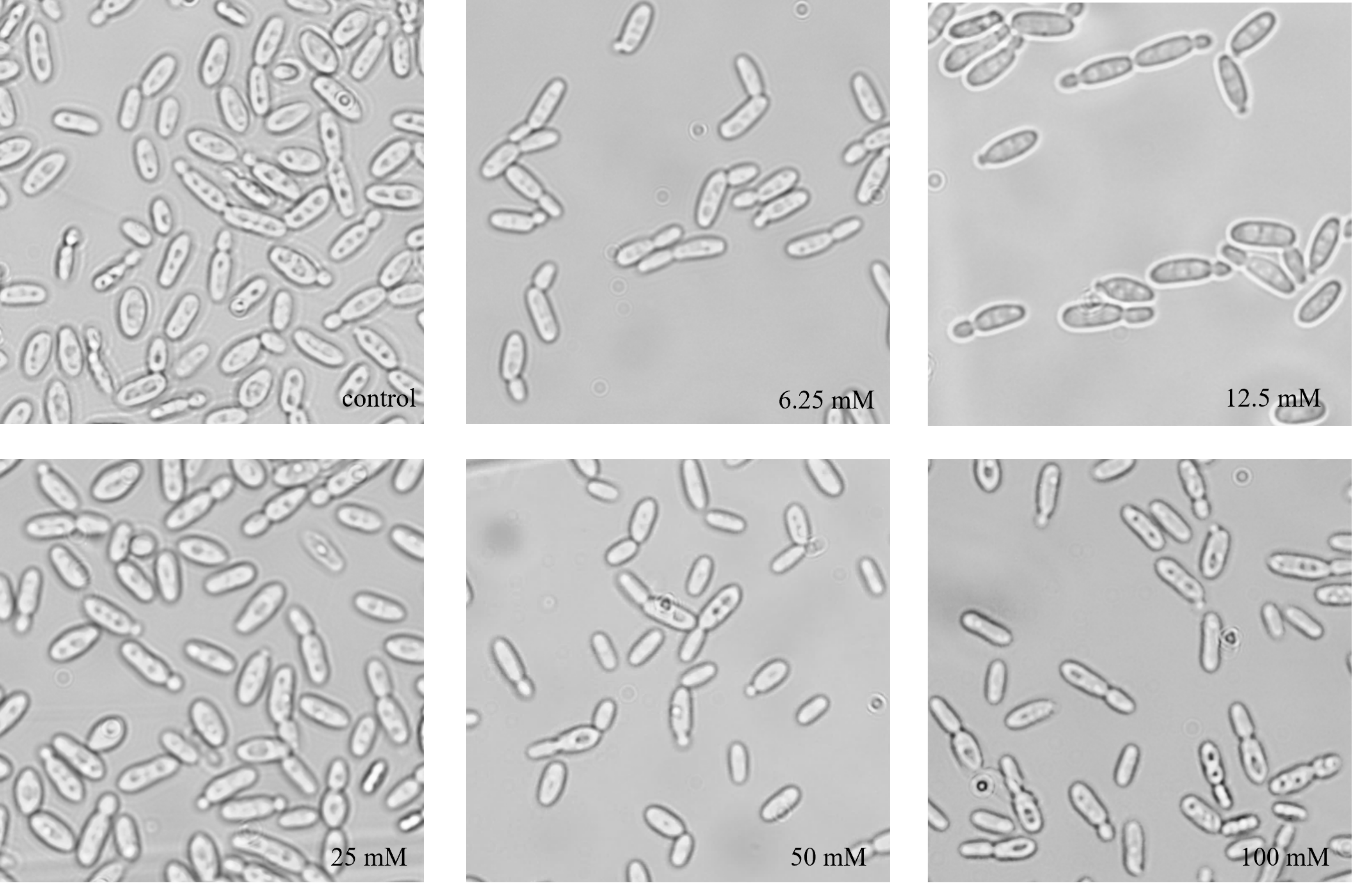
**

**Supplementary Fig. 4** Microscopy images of *Candida boidinii* at 22 °C after 24 hours of exposure to different concentrations of tyrosol (600x magnification)

**SUPPORTING INFORMATION**

**Supplementary Table 1** RMANOVA results over time to evaluate the effects of medium, and incubation temperature on the relative biofilm formation of *Candida boidinii,* bold numbers (MS, F) indicate significant differences at p < 0.05

**Supplementary Fig. 1** Relative biofilm formation of *Candida boidinii* in three different media at 22 °C (a), 30 °C (b) and 37 °C (b), data represent averages of five replicates

**Supplementary Fig. 2** Effect of increasing tyrosol concentration on CV-stained biofilm formation at 37 °C, data represent averages of five replicates

**Supplementary Fig. 3** Effect of increasing tyrosol concentration on relative biofilm formation at 30 °C (a) and 37 °C (b), data represent averages of five replicates

**Supplementary Table 2** RMANOVA results over time and temperature to evaluate the effects of tyrosol on the growth of planktonic cells of *C. boidinii*, bold numbers (MS, F) indicate significant differences at p < 0.05

**Supplementary Table 3** RMANOVA results over time and temperature to evaluate the effects of tyrosol on the biofilm formation measured with the XTT assay of *C. boidinii*, bold numbers (MS, F) indicate significant differences at p < 0.05

**Supplementary Fig. 4** Microscopy images of *Candida boidinii* at 22 °C after 24 hours of exposure to different concentrations of tyrosol (600x magnification)
